# Hyperosmolar Hyperglycemic State With Reversible Encephalopathy in a Young Female on Chronic Steroids and Opioids: A Case Report

**DOI:** 10.7759/cureus.99304

**Published:** 2025-12-15

**Authors:** Athena Myrou

**Affiliations:** 1 1st Propaedeutic Internal Medicine Department, American Hellenic Educational Progressive Association (AHEPA) University Hospital, Thessaloniki, GRC

**Keywords:** hpa axis suppression, hyperosmolar hyperglycemic state, idiopathic intracranial hypertension, insulin resistance, opioid exposure, reversible encephalopathy, steroid-induced hyperglycemia

## Abstract

Hyperosmolar hyperglycemic state (HHS) is uncommon in young adults and may signal complex metabolic and pharmacologic interactions, particularly in the context of chronic glucocorticoid and opioid exposure. We describe a 22-year-old female with morbid obesity, idiopathic intracranial hypertension treated with ventriculoperitoneal shunting and neuromodulation, chronic high-dose steroid use, and opioid exposure who presented with severe hyperglycemia, dehydration, headache, and transient confusion. Laboratory findings revealed glucose level of 583 mg/dL, calculated osmolality of approximately 318 mOsm/kg, preserved C-peptide indicating severe insulin resistance, and suppressed adrenocorticotropic hormone and cortisol consistent with exogenous steroid-induced hypothalamic-pituitary-adrenal axis suppression. Urine ketones were present, but serum ketones were absent, supporting HHS without ketoacidosis. Neuroimaging excluded acute intracranial pathology. The patient improved with intravenous fluids, insulin therapy, treatment of intercurrent infection, and careful steroid tapering, achieving full neurologic recovery. This case highlights the potential for hyperosmolar neuro-metabolic crisis in young adults with chronic steroid and opioid exposure and emphasizes the importance of considering HHS in atypical populations, particularly when obesity, prolonged glucocorticoid therapy, and central nervous system depressants coexist.

## Introduction

Hyperosmolar hyperglycemic state (HHS) is a life-threatening metabolic emergency characterized by severe hyperglycemia, hyperosmolality, dehydration, and altered mental status, typically affecting older adults with type 2 diabetes [1,2]. Its occurrence in young adults is unusual and may indicate underlying endocrine, metabolic, or pharmacologic triggers.

Chronic glucocorticoid exposure is a recognized cause of secondary diabetes and HHS due to enhanced hepatic gluconeogenesis, peripheral insulin resistance, and impaired β-cell function [3,4]. Prolonged supratherapeutic steroid use may also suppress the hypothalamic-pituitary-adrenal (HPA) axis, increasing susceptibility to metabolic instability and infection [5-7]. Opioid exposure may further obscure diagnosis by altering mental status and masking neurologic symptoms [8,9]. Idiopathic intracranial hypertension (IIH) is strongly associated with obesity [10], and intracranial neuromodulation hardware may complicate neurologic assessment and neuroimaging interpretation.

In this patient, the coexistence of IIH further complicated the clinical picture, as IIH-related symptoms such as headache and visual changes may overlap with metabolic or neurologic manifestations of HHS.

This case underscores the importance of maintaining a high index of suspicion for HHS in young adults with significant metabolic and iatrogenic risk factors and highlights the need for coordinated multidisciplinary management.

## Case presentation

A 22-year-old female with morbid obesity (BMI = 56.6 kg/m²), IIH treated with a ventriculoperitoneal shunt and occipital nerve stimulator (device model/manufacturer not documented in available records), and chronic supratherapeutic glucocorticoid exposure for presumed inflammatory disease presented to the emergency department with progressive headache, polydipsia, severe hyperglycemia, and transient confusion. Her history also included chronic opioid use with prior tramadol dependence. Her analgesic regimen consisted of long-term use of multiple opioid and centrally acting agents, including tramadol up to 450 mg/day, tapentadol (Palexia) 75 mg every 10 hours, and additional muscle relaxants (Miorel, Viloxidon) and Maroxim, taken regularly since 2021.

On arrival, she was hypertensive (180/120 mmHg) and tachycardic (113 bpm) with normal oxygen saturation and was alert and oriented without focal neurological deficits (Glasgow Coma Scale score = 15/15). Physical examination revealed cushingoid features, including centripetal obesity, violaceous striae, and edema.

Initial laboratory evaluation demonstrated serum glucose at 583 mg/dL, measured sodium at 135 mmol/L (corrected 142.7 mmol/L), urea at 70 mg/dL, creatinine at 0.82 mg/dL, and glycosylated hemoglobin (HbA1c) of 9.9%. Urine ketones were ++/+++ with absent serum ketonemia, and C-peptide was elevated (5.77 ng/mL), suggesting preserved endogenous insulin secretion and severe insulin resistance. Serum osmolality was calculated at ~318 mOsm/kg. Adrenocorticotropic hormone (ACTH) and morning cortisol were suppressed, consistent with exogenous steroid-induced HPA-axis suppression. Mild transaminitis and neutrophilic leukocytosis were noted with low inflammatory markers, likely steroid-attenuated (Table 1).

**Table 1:** Key laboratory findings on admission. Laboratory parameters on admission demonstrated severe hyperglycemia, hyperosmolality, and preserved renal and electrolyte function. Corrected sodium and effective osmolality were calculated as indicated below. * Corrected sodium: Na⁺corr = Na⁺ + 0.016 × (glucose − 100). ** Effective osmolality: 2 × Na⁺corr + glucose / 18. HHS: hyperosmolar hyperglycemic state; DKA: diabetic ketoacidosis.

Parameter	Value	Reference range	Interpretation
Glucose	583 mg/dL	74–100	Severe hyperglycemia
Sodium (measured)	135 mmol/L	136–146	Pseudohyponatremia
Sodium (corrected)*	142.7 mmol/L	—	True sodium level in HHS
Potassium	4.6 mmol/L	3.5–5.1	Normal
Urea	70 mg/dL	17–43	Dehydration/pre-renal
Creatinine	0.82 mg/dL	0.66–1.9	Normal
Glycosylated hemoglobin (HbA1c)	9.9%	4.0–6.0	Chronic dysglycemia
C-peptide	5.77 ng/mL	1.1–4.4	Severe insulin resistance
Urine ketones	++/+++	Negative	No DKA
Serum osmolality (calculated)**	~318 mOsm/kg	275–295	Hyperosmolar state

**Figure 1:**
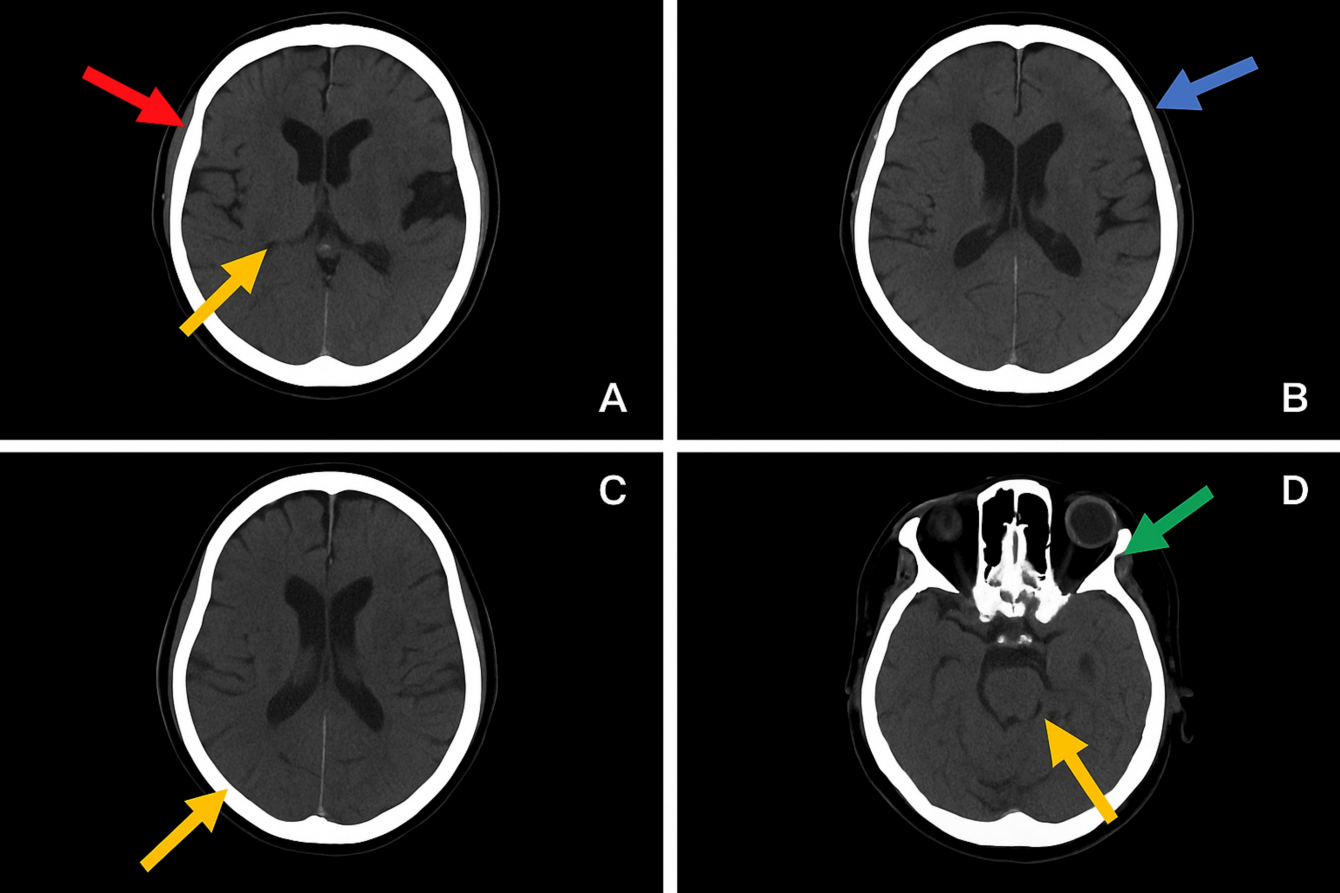
Non-contrast CT of the brain. Axial non-contrast CT images of the brain (A–D) demonstrating no intracranial hemorrhage, no abnormal parenchymal densities, and no midline shift. The ventricular system and subarachnoid spaces appear within normal limits. Mild widening of the subarachnoid space at the vertex is noted. A partially empty sella is present. Several ethmoidal air cells show opacification, while the remaining paranasal sinuses are well aerated. Impacted maxillary teeth projecting into the maxillary sinus floor are visualized. Linear hyperdense subcutaneous structures in the occipital region create streak artifacts. A: Red arrow = widening of the subarachnoid space at the vertex; yellow arrow = partially empty sella. B: Blue arrow = prominent cortical sulci. C: Yellow arrow = prominence of subarachnoid space. D: Green arrow = opacification of ethmoidal air cells; yellow arrow = impacted maxillary tooth projecting into the maxillary sinus floor.

**Figure 2:**
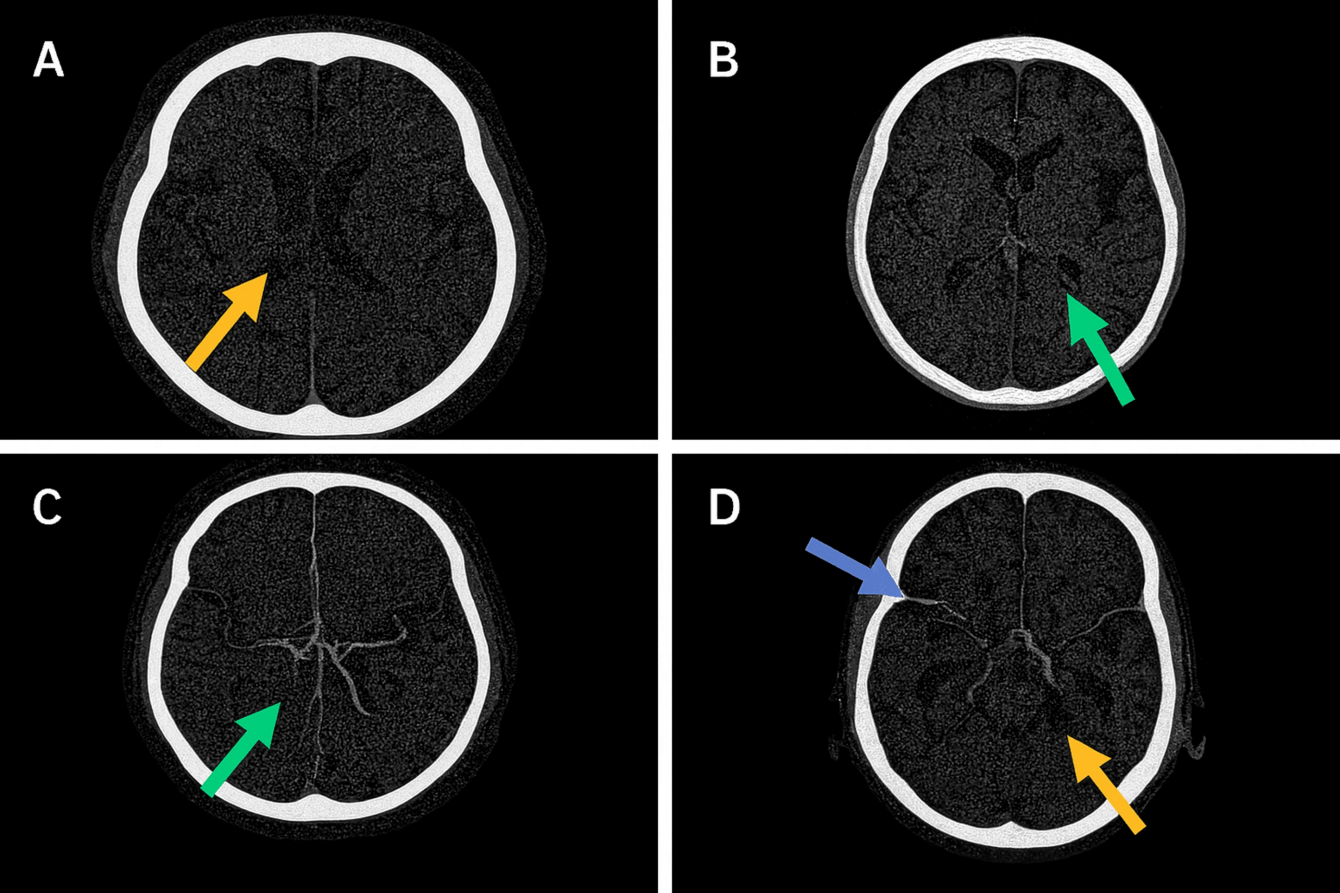
Axial CT angiography images of the brain. A: Normal opacification of the anterior circulation, with symmetric filling of the distal internal carotid arteries and proximal middle cerebral arteries (yellow arrow). No arterial stenosis or occlusion is present. B: The middle cerebral artery branches demonstrate normal contrast enhancement without evidence of vascular narrowing or filling defects (green arrow). C: Continued visualization of patent middle cerebral artery branches with preserved caliber and branching pattern (green arrow). D: Normal opacification of the posterior circulation, including the basilar artery and posterior cerebral arteries (yellow arrow). The superficial cortical veins over the convexity also appear normally opacified (blue arrow). No aneurysm, arteriovenous malformation, or venous thrombosis is identified.

Neuroimaging with non-contrast CT and CT angiography/venography revealed no acute intracranial pathology or venous thrombosis. Imaging quality was partly limited by the neuromodulation device. No clinical or radiologic evidence of shunt malfunction was identified (Figures 1, 2).

Management and clinical course

The patient was diagnosed with HHS without ketoacidosis and was treated with aggressive isotonic fluids, intravenous insulin, electrolyte management, and continuation of glucocorticoids with a cautious taper to avoid adrenal crisis. During hospitalization, she experienced a brief episode of transient encephalopathy that resolved spontaneously without new findings on repeat imaging. A transient bacteremia was identified and treated according to culture sensitivities.

She received high-dose intravenous hydrocortisone in multiple daily doses over a 13-day period, followed by gradual tapering according to clinical response.

Her neurologic and metabolic status progressively improved. She was transitioned to basal insulin (glargine) with correctional rapid-acting insulin and discharged with close endocrine and neurology follow-up. Written informed consent was obtained from the patient for publication of this case.

## Discussion

This case illustrates the interplay of metabolic, endocrine, neurologic, and pharmacologic factors leading to HHS in a young adult. Morbid obesity, prolonged supratherapeutic glucocorticoid exposure, and chronic opioid use produced a high-risk environment for metabolic decompensation [11,12]. The markedly elevated C-peptide level indicated profound insulin resistance rather than insulin deficiency, consistent with steroid- and obesity-driven mechanisms [3,4].

Glucocorticoids are frequently implicated in hyperglycemic crises due to their effects on gluconeogenesis, lipolysis, and peripheral insulin sensitivity [3,4]. Long-term exposure additionally suppresses the HPA axis, blunts inflammatory responses, and predisposes patients to infection and metabolic deterioration [5-7]. Obesity further amplifies systemic inflammation, worsens insulin resistance, and increases vulnerability to hyperglycemic emergencies [13].

Neurologic manifestations of HHS are mediated by hyperosmolality, dehydration, and electrolyte imbalance [2,10]. In this case, opioid exposure is likely to have contributed to fluctuating mental status and confounded neurologic assessment [14,15]. Despite ketonuria, the absence of serum ketonemia, preserved acid-base status, and normal bicarbonate levels made diabetic ketoacidosis (DKA) unlikely [2,16]. In contrast, the patient’s markedly elevated effective osmolality (~318 mOsm/kg) met the diagnostic threshold for HHS as defined by current American Diabetes Association (ADA) and Joint British Diabetes Societies for Inpatient Care (JBDS) guidelines [2,10]. These parameters, together with preserved endogenous insulin secretion, strongly favored the diagnosis of HHS over DKA. The presence of bacteremia in the setting of low inflammatory markers exemplifies the immunosuppressive effects of chronic corticosteroid therapy [17].

Opioid exposure may have contributed to the patient’s transient encephalopathy through several mechanisms. Activation of μ-opioid receptors produces central nervous system (CNS) depression, blunts arousal pathways, and can impair attention and vigilance even at therapeutic doses [18]. In the context of hyperosmolarity, neuronal dehydration and osmotic shifts may further reduce cortical excitability, potentially amplifying opioid-related suppression of consciousness [19]. The combination of μ-receptor-mediated CNS depression and hyperosmolar metabolic stress, therefore, provides a plausible explanation for the patient’s transient alteration in mental status.

Management aligned with HHS guidelines, emphasizing careful fluid resuscitation, intravenous insulin administration, electrolyte monitoring, and continuation of glucocorticoids with a controlled taper to avoid adrenal crisis [2,10]. The reversible encephalopathy observed here highlights the importance of early recognition and prompt metabolic correction.

This report has several limitations inherent to single-patient case studies, which limit the generalizability of the observations. Medication adherence, including the timing and dosage of opioid intake, could not be fully verified because documentation relied partially on patient self-report. Consequently, the temporal relationship between opioid exposure and the brief episode of encephalopathy remains uncertain. Additionally, incomplete historical records regarding the neuromodulation device and long-term steroid use constrain the precision of mechanistic interpretation. Despite these limitations, the case provides valuable clinical insights into the complex metabolic and neurologic interactions in young adults with significant iatrogenic and pharmacologic risk factors.

## Conclusions

The present case highlights an uncommon occurrence of HHS in a young adult without known diabetes, precipitated by severe insulin resistance from morbid obesity and prolonged supratherapeutic glucocorticoid exposure, with additional contribution from opioid-associated central nervous system vulnerability. Early recognition of the hyperosmolar state, despite the presence of ketonuria, was essential in differentiating HHS from DKA and guiding appropriate therapy. The coexistence of steroid-induced metabolic dysregulation, HPA-axis suppression, and blunted inflammatory responses created a permissive environment for metabolic crisis and transient encephalopathy. Opioid exposure further complicated neurologic assessment and may have contributed to fluctuating mental status during hospitalization. Prompt fluid resuscitation, insulin therapy, infection management, and a carefully controlled steroid taper facilitated full neurological and metabolic recovery. This case underscores the importance of maintaining a high index of suspicion for HHS in young adults with overlapping endocrine, pharmacologic, and iatrogenic risk factors, and highlights the critical role of multidisciplinary management in optimizing outcomes.
